# Short-Term Withdrawal of Mitogens Prior to Plating Increases Neuronal Differentiation of Human Neural Precursor Cells

**DOI:** 10.1371/journal.pone.0004642

**Published:** 2009-02-27

**Authors:** Telma Tiemi Schwindt, Fabiana Louise Motta, Gabriela Filoso Barnabé, Cristina Gonçalves Massant, Alessander de Oliveira Guimarães, Maria Elisa Calcagnotto, Fabio Silva Conceição, João Bosco Pesquero, Stevens Rehen, Luiz E. Mello

**Affiliations:** 1 Department of Physiology, Universidade Federal de São Paulo (UNIFESP), São Paulo, Brazil; 2 Department of Biophysics, Universidade Federal de São Paulo (UNIFESP), São Paulo, Brazil; 3 Instituto de Ciências Biomédicas, Universidade Federal do Rio de Janeiro, Rio de Janeiro, Brazil; University of Sydney, Australia

## Abstract

**Background:**

Human neural precursor cells (hNPC) are candidates for neural transplantation in a wide range of neurological disorders. Recently, much work has been done to determine how the environment for NPC culture *in vitro* may alter their plasticity. Epidermal growth factor (EGF) and fibroblast growth factor-2 (FGF-2) are used to expand NPC; however, it is not clear if continuous exposure to mitogens may abrogate their subsequent differentiation. Here we evaluated if short-term removal of FGF-2 and EGF prior to plating may improve hNPC differentiation into neurons.

**Principal Findings:**

We demonstrate that culture of neurospheres in suspension for 2 weeks without EGF-FGF-2 significantly increases neuronal differentiation and neurite extension when compared to cells cultured using standard protocols. In this condition, neurons were preferentially located in the core of the neurospheres instead of the shell. Moreover, after plating, neurons presented radial rather than randomly oriented and longer processes than controls, comprised mostly by neurons with short processes. These changes were followed by alterations in the expression of genes related to cell survival.

**Conclusions:**

These results show that EGF and FGF-2 removal affects NPC fate and plasticity. Taking into account that a three dimensional structure is essential for NPC differentiation, here we evaluated, for the first time, the effects of growth factors removal in whole neurospheres rather than in plated cell culture.

## Introduction

Evidence of neurogenesis in the adult brain of birds [1], rodents and primates[2], [3], [4], and the demonstration of the presence of stem cells in specific brain regions, such as the subventricular zone (SVZ) and the hippocampus [5], [6], brought new perspectives for cell therapy and neural regeneration [7]. During development of the central nervous system (CNS), there is extensive proliferation of neuroepithelial cells lining the ventricular walls which give rise to the neurons, astrocytes and oligodendrocytes of the mature brain [8]. An experimental model to study neural stem cells is the heterogeneous free-floating aggregates of cells, termed neurospheres [9], [10], [11]. Each neurosphere is derived from a single stem cell that, by asymmetrical division, gives rise to another stem cell and one progenitor cell. The progenitor cells, in turn, give rise only to other progenitor cells. In this way, only a small fraction of the neurosphere corresponds to genuine stem cells [12]. Here we use the terminology neural precursor cells (NPC) to describe both cell types within the neurosphere [13], [14].

Despite the advances in stem cell studies, comparatively less effort has been devoted to determine the ideal culture medium for NPC expansion *in vitro*. Neurospheres are usually cultured in medium containing the mitogens FGF-2 and EGF [15]. Acquisition of EGF responsiveness by neural precursor cells is promoted by FGF-2 in the early development *in vitro* [15]. Extensive evidence has shown that EGF and FGF-2 promote proliferation while retaining the cells in an undifferentiated state [16], [17].

We suggest that the removal of growth factors EGF and FGF-2 from the medium provokes the neurospheres to start the differentiation process even in suspension. Here we tested the hypothesis that the removal of these mitogens in whole neurospheres prior to plating influences the plasticity of human NPC.

## Results

### hNPC Proliferation Profile

**Figure 1:**
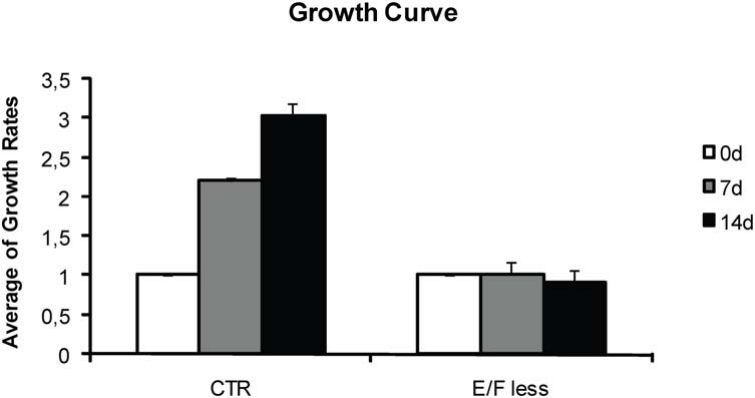
Growth curve of hNPC cell line. Single human neurospheres were isolated in wells and cultured for 14 d in the presence (CTR) or absence (MFM) of EGF and FGF-2 (n = 5). While the average growth rates of CTR group significantly increases with time in culture, MFM neurospheres do not grow (ANOVA, Newman-Keuls post hoc test). The graphic is a representation of M031 CX cell line. M046 CX cell line behaviored in the same way.

**Figure 2:**
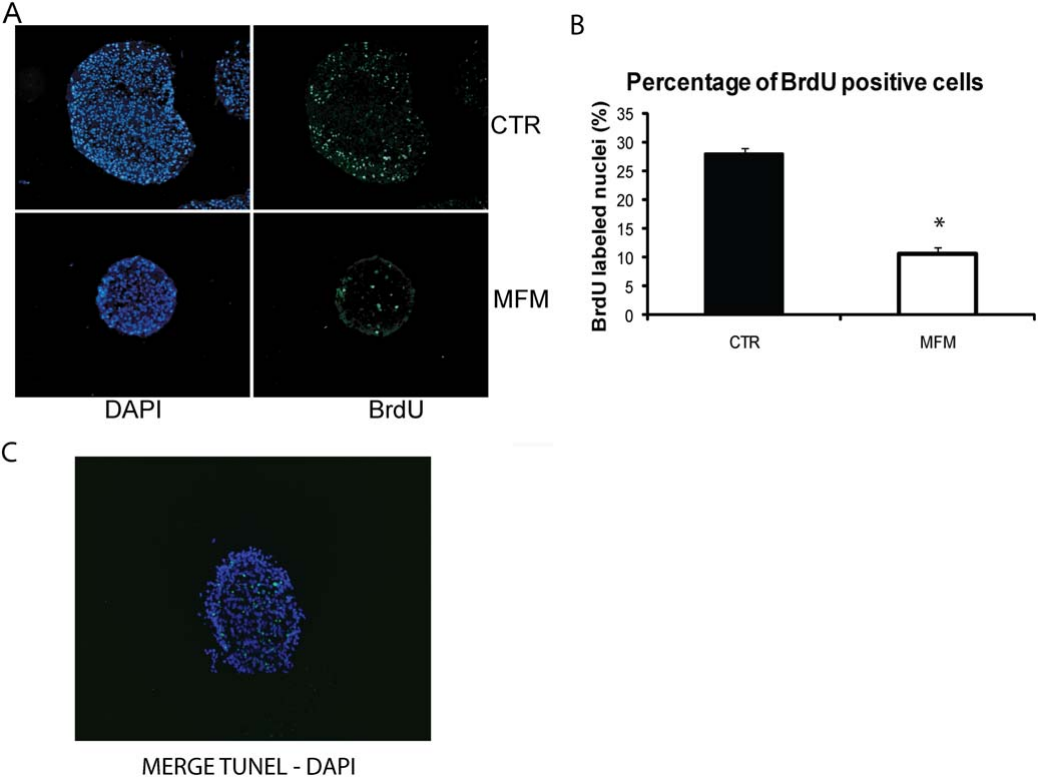
Cell proliferation before and after growth factor starvation. CTR group of hNPC showed BrdU positive cells (green) in the borders of the neurosphere, while MFM group showed very few positive cells distributed in the neurosphere. Total nuclei were stained with DAPI (blue). 200× magnification (A). Graphic shows the percentage of BrdU positive cells for CTR and MFM groups of hNPC. Note the significant decrease in the percentage of proliferating cells in the MFM group compared with CTR (n = 10, t-Student-test, p<0.05) (B). TUNEL labeled cells were concentrated in the core of the neurosphere (C), and apoptosis increased slightly after EGF and FGF-2 removal.

NPC from human fetal cortices (hNPC) were cultured in the presence or in the absence of EGF and FGF-2 for 14 days. To verify basic aspects of neurosphere growth we performed growth rates (figure 1), BrdU and TUNEL assays (figure 2), comparing the control (CTR) group and the group cultured without EGF and FGF-2, in mitogen free medium (MFM). According to the growth rates, neurospheres cultured in the presence of EGF and FGF-2 (CTR) have the volume increased three times after 14 d in culture, and they go from a 0,2 mm to about 1 mm large. For the neurospheres grown in the absence of EGF and FGF-2, their size did not change and, after 14 d, they were about 0.2–0.3 mm large (figure 1). As expected, counts of BrdU labeled cells showed that growth factors removal decreased cell proliferation (27.9±1.03% of BrdU positive cells for the CTR to 10.5±1.12% for the MFM group) (figure 2A, B). BrdU data support the reduced growth rates, but it is also important to verify the apoptosis rates, once the size of a neurosphere is given by a balance between proliferation and cell death. TUNEL experiments showed an increase in the number of apoptotic cells for both the CTR and MFM groups along 14 d in culture. However CTR group increased the percentage of apoptotic cells from 0.2±0.03% to 2.5±0.41%, while the MFM group increased the percentage of apoptotic cells from 0.8±0.05% to 5.5±1.47%. This observation means that, after 14 d in culture, the MFM group showed significant higher levels of apoptosis.

### Localization of β-tubulin III, GFAP and Nestin positive cells in whole neurospheres is modified by removal of growth factors

**Figure 3:**
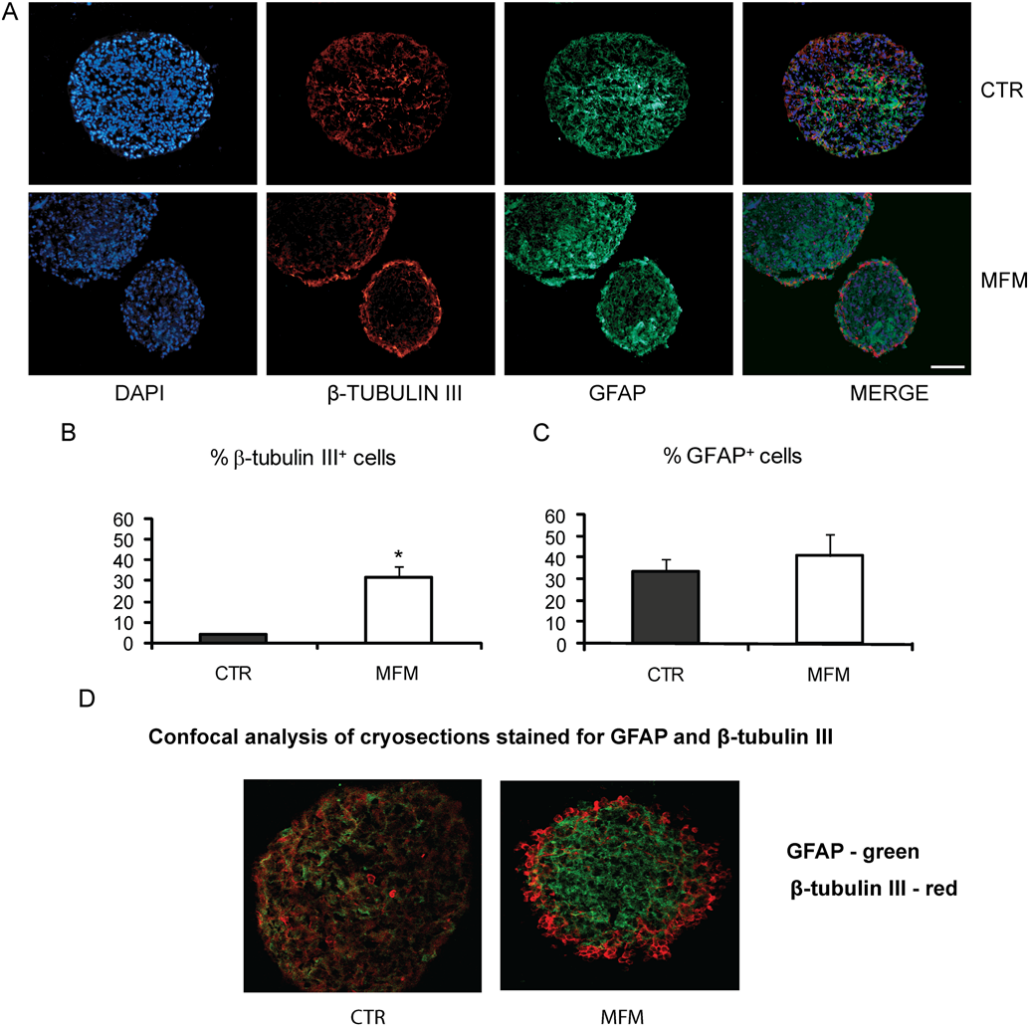
Immunolabeling of hNPC slices for β-tubulin III positive and GFAP positive cells from CTR and MFM groups. GFAP positive cells (green) were found across the whole neurosphere whereas β-tubulin III positive cells (red) were concentrated in the neurosphere core of CTR neurospheres. In the MFM group we can see β-tubulin III positive cells in the borders, without modification in GFAP localization (A). Graphic shows the percentage of β-tubulin III (B) and GFAP (C) positive cells for CTR and MFM groups of hNPC. Note the increase in the percentage of β-tubulin III positive cells in the MFM group compared with CTR (n = 10, t-Student-test, p<0.05). Confocal analysis of cryosections stained for GFAP and β-tubulin III showed that only a very small number of cells within the neurosphere co-label for GFAP and β-tubulin III (D).

**Figure 4:**
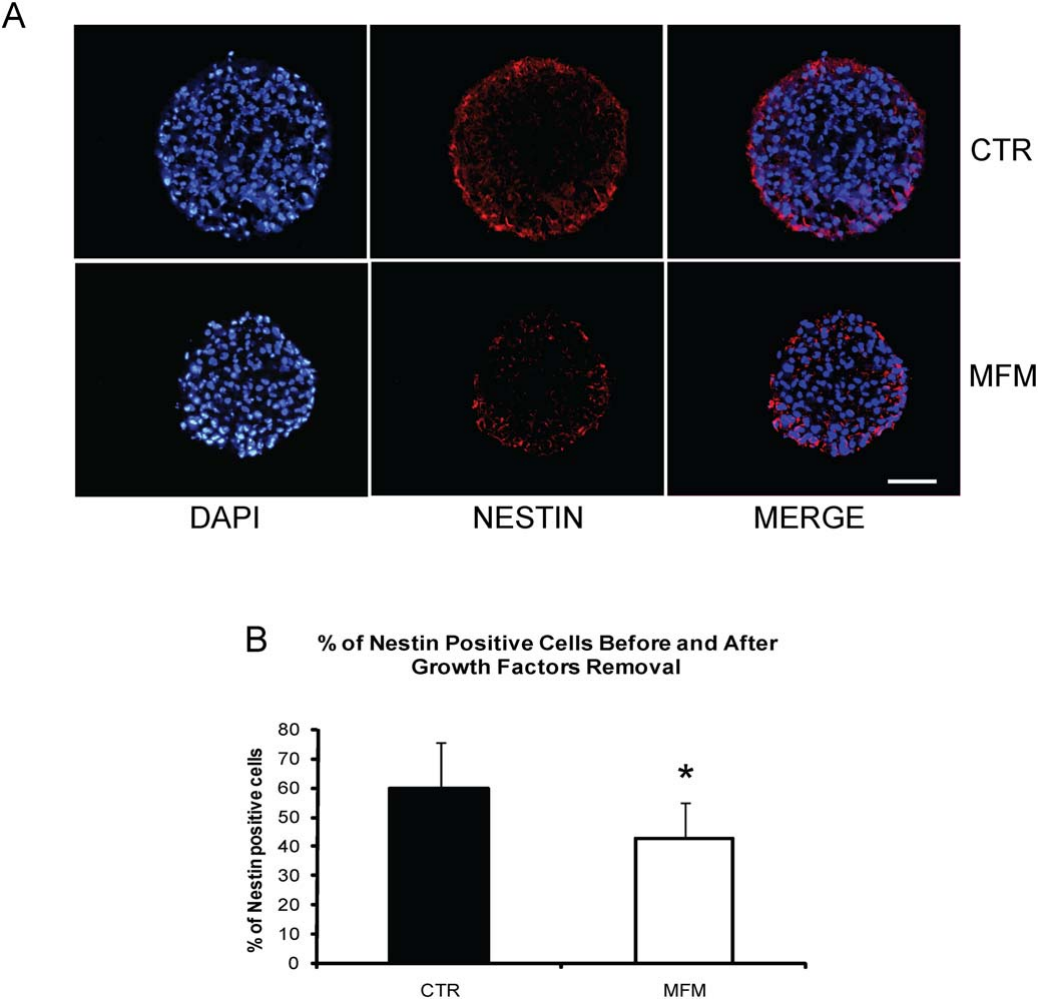
Nestin immunolabeling of hNPC cultured with or without EGF and FGF-2. Nestin positive cells (red) were found mainly in the borders of neurosphere in both CTR and MFM groups (A) Graphic shows the percentage of Nestin positive cells for CTR and MFM groups (B) of hNPC. Note the higher percentage of Nestin positive cells in the CTR group compared with MFM (B) (n = 10, t-Student-test, p<0.05).

Ten micrometer thick cryosections of hNPC were used to evaluate the distribution of the four main neural populations (β-tubulin III, GFAP, Gal-C and Nestin positive cells) across the whole spheres. The analyzed hNPC cryosections were absent for Gal-C positive cells (data not shown). Staining of hNPC sections for β-tubulin III and GFAP yielded a different distribution and localization between the MFM and CTR groups. For hNPC from the CTR group, GFAP was found across the whole neurosphere, whereas β-tubulin III positive cells were concentrated in the core of the neurosphere (figure 3A). However, after growth factors removal, GFAP was found in the whole section and β-tubulin III positive cells were found in the borders of neurospheres (figure 3A). Even the localization of GFAP positive cells did not change, the intensity of GFAP staining seemed to be higher in the areas where β-tubulin III positive cells concentrate (core for the CTR and borders for the MFM groups). Then, we checked by confocal microscopy analysis whether hNPC could express both GFAP and β-tubulin III simultaneously. We found that only a very small proportion of cells in the neurosphere in both conditions (CTR and MFM) co-labeled for both GFAP and β-tubulin III (approximately 1 co-localization per sphere section) (figure 3D), what seemed to be irrelevant to characterize NPC fate. Besides the changes found in the localization of β-tubulin III and GFAP positive cell populations in the neurospheres, we also investigated whether the potential of hNPC in suspension was also affected quantitatively by growth factors withdrawal. Interestingly, we found 3.65±0.52% of β-tubulin III and 33.51±5.78% GFAP positive cells for the CTR group, while the MFM group gave rise to a significantly (t-Student-test, p<0.05) greater proportion of β-tubulin III (31.72±4.80%) and GFAP positive cells (41.16±10.18%) (figure 3B, C). Since the proportion of more committed progenitor or differentiated cells (represented by β-tubulin III and GFAP positive cells) was increased in MFM condition, it is plausible to find a reduction in the proportion of multipotent neural stem cell population. We used Nestin as a marker for undifferentiated hNPC. Nestin was localized in the borders of the sphere for both the CTR and MFM groups (figure 4A), but the percentage of Nestin positive cells changed from 59.9±15.4% to 42.30±12.73% after EGF and FGF-2 removal (t-Student-test, p<0.05) (figure 4B). These data indicate that the number of undifferentiated cells decreased after 14 d in culture in the absence of EGF and FGF-2.

### Growth factors withdrawal induces changes in gene expression profiles in whole human NPC

**Figure 5:**
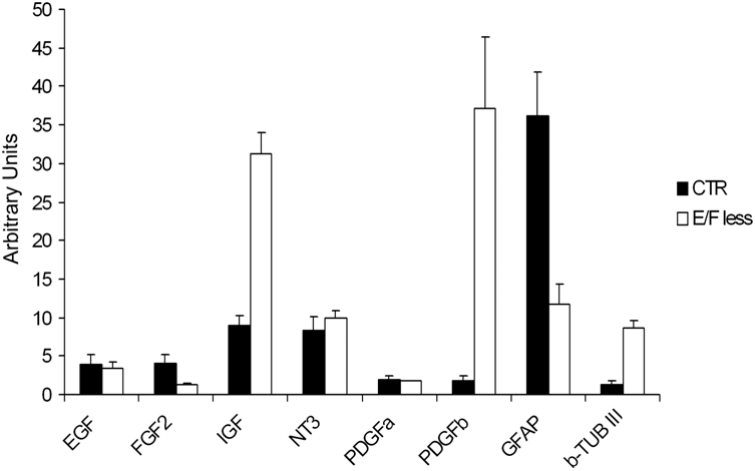
Gene expression profile before and after growth factor starvation in hNPC. After 14 days growth factors withdrawal, hNPC decreased the expression of *fgf-2* and *gfap* and increased the expression of *igf-1* and *pdgfb*. hNPC also showed an increase in *β-tubulin III* expression. We show the mean relative expression of two independent reactions calculated as described in methods with the propagated Cq standard deviations. Primer sequences are listed in Table S1.

We used real-time PCR technique to evaluate changes in gene expression in the hNPC CTR and MFM groups. The expression of a number of growth factors that are relevant for cell proliferation and survival (*egf*-Epidermal Growth Factor, *fgf-2*– Fibroblast Growth Factor 2, *igf-1*– Insulin-like Growth Factor 1, *nt3* – Neurotrophin 3, *pdgfa* and *pdgfb* – Plateled Derived Growth Factor a and b, as well as neural markers, such as *gfap* and *β-tubulin III*) was measured using real time-PCR technique (for primer sequences see Table S1). Results showed that, after 14 days of growth factors withdrawal, hNPC decreased the expression of *fgf-2* and *gfap* and increased the expression of *igf-1*, *pdgfb* and *β-tubulin III* as measured by real time-PCR (figure 5). Our data are in agreement with the findings of Einstein and colleagues [18] and Campos and colleagues [19] for mouse NPC (mNPC). To more fully appreciate the relation of our human data to murine data we also used three independent cultures of mNPC in real time-PCR assays to evaluate gene expression before and after growth factors removal (for primer sequences see Supplemental table S1, Supplemental figure S1). The obtained results were very similar to that observed for hNPC. We found that, after 10 days of growth factors withdrawal, mNPC decreased the expression of *fgf-2*, *gfap* and also *nestin* and increased the expression of *igf-1* and *pdgfb*. For *β-tubulin III*, despite the lack of statistically significant different expression between the CTR and MFM groups, there was a clear tendency of an increase in the expression of this protein after growth factors removal.

### Neurospheres cultured in suspension under growth factors starvation condition give rise to neurons with longer processes

**Figure 6:**
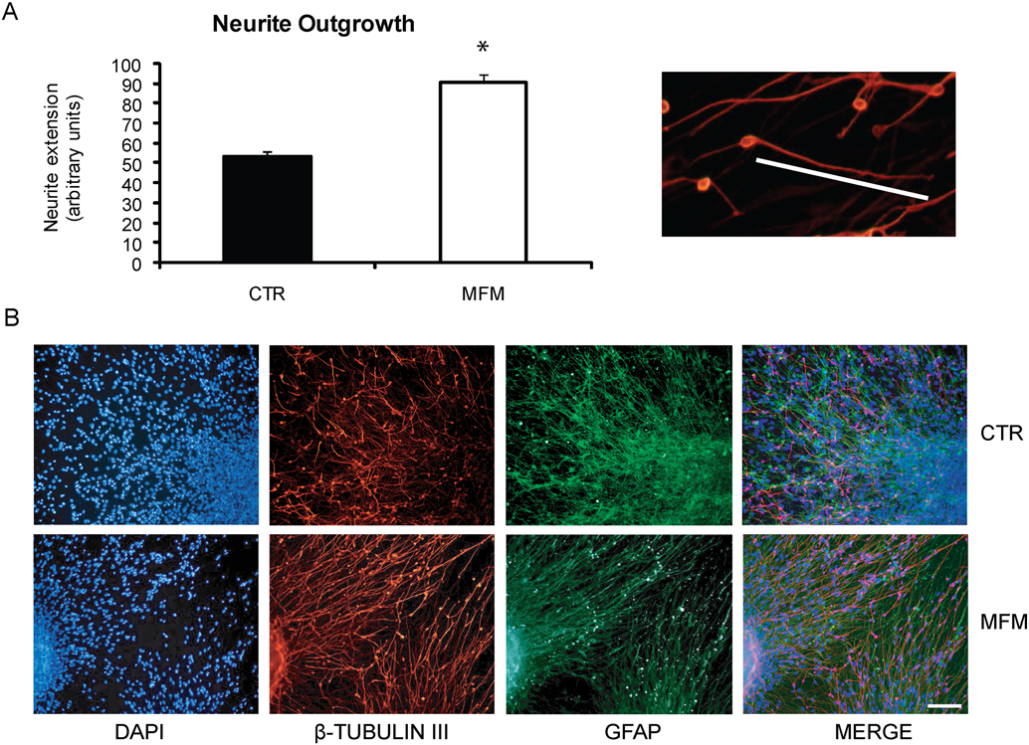
Growth factor removal promotes neurite extension in hNPC. Immunocytochemistry for β-tubulin III showed neurons with longer processes in the MFM group, while the CTR group originated neurons with shorter processes (t-Student-test, p<0.05) (A). Higher magnification images of the differentiated neurons were used to access neurite extension after plating and migration, considering the distances between the cell bodies and the end of the longer processes found (n = 15). Immunolabeling for β-tubulin III+(red) and GFAP+cells (green) for the CTR and MFM after plating and 7 days differentiation. The processes were longer in the MFM group, as well as the radial orientation, that was absent in the CTR group (B). Total nuclei were stained with DAPI (blue). 200× magnification.

hNPC were plated onto coverslips (approximately 5 neurospheres per coverslip) to allow migration and neural differentiation of cells. To verify whether growth factors starvation could change the capability of cells to migrate, we measured the radius of their migration spread. The analysis showed no differences in migration between the CTR and MFM groups. Immunocytochemistry for β-tubulin III in plated neurospheres after mitogens removal (MFM) originated processes (dendrites)whose extension was twice longer than that observed for the CTR group (t-Student-test, p<0.05, figure 6A). The orientation of the β-tubulin III and GFAP positive cells also seemed to be altered by growth factors starvation prior to plating. Before starvation, processes lacked a clear orientation in relation to the neurosphere, whereas after starvation a substantial number of processes could be seen radially oriented from the neurospheres (figure 6B). No evidence of the oligodendrocyte lineage was found, due to the lack of Gal-C positive cells after hNPC differentiation (data not shown).

## Discussion

The main findings of this work were that the short-term removal of growth factors is capable of (i) modifying the distribution of β-tubulin III and GFAP positive cells in whole neurospheres; (ii) increasing the number of β-tubulin III positive cells, while decreasing the number of Nestin positive cells (iii) inducing changes in gene expression profiles; and (iv) promoting neurite extension and changes in orientation of neural stem cells.

Here we show the ability of hNPC to survive and differentiate after culturing in suspension in the absence of growth factors. It has recently been shown that mNPC are able to survive and differentiate after 14 days growth factors starvation [18]. However, MFM neurospheres did not grow as much as the CTR ones, as observed in growth rates (figure 1). As neurosphere size is established by the balance between proliferation and cell death, the stable size of MFM neurospheres is due to both proliferation and apoptosis decreasing (figure 2). As expected, BrdU incorporation decreases after mitogens removal (27.9% for CTR; 10.5% for MFM). On the other hand, we have to take in account that, even after growth factors removal, the level of apoptotic cells was extremely low (about 5%), again, showing that neurospheres should have a mechanism that prevents cell death in this condition. The increased apoptosis after 14 days in culture in the CTR group can be attributed to the increase in neurosphere size. As neurospheres grow, the cells from the core are exposed to lower concentration of growth factors, leading to an increased cell death (figure 2C). This may be due to the fact that neurospheres deprived of growth factors in suspension are able to produce cytokines and growth factors that, at the same time, can induce differentiation and prevent cell death.

It could be argued that, owing to the fact that EGF and FGF-2 are known to induce self-renewal of NPC, the effects found after removal of these factors on cell differentiation is predictable and expected based on published literature. Yet, Caldwell and colleagues [20] showed that the combination of cell–cell interactions during differentiation and growth factor administration, can increase the number of generated neurons . This is only one example of the importance of cell-cell interaction and of the aspects associated to its three-dimensional structure for neurosphere plasticity.

Staining for GFAP, β-tubulin III and Nestin in hNPC from the CTR and MFM groups cultured in suspension revealed two major findings. Removal of growth factors modifies the distribution and proportions of β-tubulin III, GFAP and Nestin positive cells in whole neurospheres. In the CTR group, GFAP (34%) was found across the whole neurosphere, β-tubulin III (4%) was preferably expressed in the neurosphere core (figure 2A, B) and Nestin (60%) was found in the border of the sphere (figure 4A). The cell distributions for the CTR group are in agreement with the findings of Campos and colleagues [19] for a three-dimensional model of mouse neurospheres. In the MFM group, β-tubulin III positive cells (32%) concentrated in the border of the neurospheres, while GFAP (41%) and Nestin (42%) positive cells kept the same localization. (figure 3A). We highlight the fact that Nestin localization in the CTR group was the same as that of BrdU positive cells. Despite Nestin localization was not altered, it is important to notice that the percentage of positive cells significantly decreases, explaining the increased differentiation in the borders of the neurosphere after mitogens removal. *β-tubulin III* expression increased in hNPC (figure 5) and displayed a clear tendency of increasing in mNPC (Supplemental figure S1), after growth factors removal, as showed by real-time PCR .Together, these results reinforce the hypothesis that mitogens removal, even without adhesion and migration, is responsible for an increased cell differentiation.

We hypothesize the shift in the distribution of β-tubulin III positive cells was caused by a decreased gradient of growth factors from the outer layer to the center of neurospheres. Given that the concentration of EGF and FGF-2 inside the neurosphere might be lower than in the outside (in spheres cultured in the presence of these mitogens), cells in the neurosphere core are able to stop proliferation and start differentiation even in suspension. It is reasonable to assume that cells in the inner portion of the neurosphere would more readily be deprived from growth factors and this, in turn, would trigger the endogenous production of growth factors, initially or mostly from the neurosphere core. Growth factors production by the core would be responsible for maintaining an undifferentiated state in the core while setting cells with neuronal markers in the borders of the neurosphere (that lack growth factors). In the core of the MFM groups, the smaller concentrations of such autocrine production of growth factors, as compared to exogenous administration in the medium, would be sufficient to maintain an undifferentiated state while insufficient to trigger mitotic activity. This represents a logical and possible hypothesis for explaining the altered distribution of β-tubulin III positive cells (core *versus* shell) as a consequence of growth factors withdrawal. Taken together, these results show that, during growth factors removal, there is a significant shift in the expression of growth factors and neural specific markers (*gfap*, *β-tubulin III* and *nestin*) by NPC. It was already shown that mouse neurosphere cells deprived of growth factors are able to produce PDGF and FGF-2 among other factors [18]. However, for human neurospheres that had not been described yet. Our Real Time-PCR results showed that hNPC from the MFM group had decreased expression of *fgf-2* and *gfap* and increased expression of *igf-1* and *pdgfb* (figure 5). This, in part, could explain the differences in the level of differentiation and in β-tubulin III positive cells distribution in the neurospheres before and after growth factors removal.

Immunocytochemistry assays showed that, after 7 days plating, hNPC differentiated into β-tubulin III and GFAP positive cells, but no oligodendrocytes for both the CTR and the MFM groups. Lack of oligodendrocytes after human neurosphere differentiation (plated or in suspension) must be attributed to the absence of proliferation of true stem cells *in vitro*, as reported by Wright and colleagues [21]. We demonstrate that human cell lines in the MFM group showed neurons with longer processes, while the CTR group originated neurons with shorter processes. The longer processes found after mitogens starvation can be explained by the fact that some growth factors that affect neurite extension are up regulated. For instance, PDGFb and IGF-1 promote neurite outgrowth [22], [23], [24], [25]. Given our finding of a significant increase in those growth factors expression after mitogens removal, we suggest that PDGFb and IGF-1 could be partially responsible for neurite extension in the MFM group. In addition, before starvation, processes lacked a clear orientation in relation to the neurosphere, while after starvation a substantial number of processes could be seen radially oriented from the neurospheres.

These features could be relevant for priming the cells just before implantation in a different environment in the course of regenerative strategies. Indeed, altering the conditions to which NPC are subjected just prior to being implanted in an injured structure might be critical for defining its potential for survival and integration. Here we did not evaluate whether or not this potential translates to the same effect *in vivo*. Yet, our data prompt us to hypothesize that growth factors starvation prior to NPC transplantation might enhance the ability of these cells to graft and provide functional recovery.

We also suggest that growth factors starvation prior to NPC transplantation might enhance the ability of these cells to graft and provide functional recovery. The observations concerning the organized sprouting of astrocytes and neurons after growth factors withdrawal could result in a better targeting of the transplanted cells to the lesion site. Rather than providing optimal surviving conditions for cultures, we hypothesize that adjusting culture parameters (including growth factors withdrawal) might be essential for achieving success when grafting these cells in damaged systems. Similar to ischemic cell conditioning where pre-exposure of neural cells to brief ischemic episodes render these cells rather resistant to subsequent ischemia [26], it is possible that transitory withdrawal of EGF and FGF-2 might trigger the expression of specific gene programs for differentiation and/or neuroprotection. The differences found in neuronal and glial differentiation between the CTR and MFM groups, give rise to a new question: Can growth factors removal influence the capability of migration, integration and differentiation of NPC after cell transplantation? This is an important issue to be evaluated and could be relevant for the functional recovery of neurological disorders.

## Materials and Methods

### Culturing human NPC

M031 CX and M046 CX cell lines of neurospheres at passage 8 and 7, respectively, were obtained from the Stem Cell Research Program at the Waisman Center, University of Wisconsin, Madison, USA. The collection method was in agreement with the rules of the National Institutes of Health for tissue collection, and it was performed at the University of Wisconsin, Madison. Human fetal brain cortices were obtained from two individuals, M031 CX (80 days post-conception) and M046 CX (87 days post-conception). After dissection, cells were seeded into growth medium consisting of Dulbecco's modified eagle medium (DMEM)/F12/penicillin-streptomycin-amphotericin (PSA) containing B27, EGF, FGF-2, and heparin at a density equivalent to 100,000cells/mL. When most of the human neurospheres reached approximately 1 mm diameter, all of them (large and small ones) were passaged by sectioning of spheres with a Mc Ilwain Tissue Chopper into 200 µm sections and re-seeded into fresh growth medium. B27 is a supplement that was previously shown to support more neuronal survival and proliferation [13]. The tissue was stored as frozen neurospheres and shipped as such when requested (such as in the current experiment). The cells were never able to generate oligodendrocytes, neither in the laboratory where they were obtained (Waisman Center) nor in our current experiments. Half of the medium was replaced every 4 days and passaging of cells was undertaken every 10–14 days. For 5-bromo-2′deoxyuridine (BrdU) incorporation and differentiation of plated neurospheres, cells were at passage 11 (P11); for differentiation in suspension cells were at passage 19 (P19).

### Ethical issues

This work was developed under the approval of the Ethics Committee of Universidade Federal de São Paulo (UNIFESP), process 0976-04. The fetal tissue was collected from an NIH supported tissue bank that conformed to current US guidelines on informed consent and with approval by the University of Wisconsin Insitutional Review Board (IRB).

### Growth factors withdrawal

The M031 CX and M046 CX spheres were transferred to conical tubes and washed carefully 3 times with 8 mL pre warmed DMEM. If necessary, the spheres were centrifuged at 900 rpm. The spheres were put in growth factors free medium (DMEM/F12/B27) and kept in those conditions in suspension for 10 and 14 days, for mouse and human cells, respectively. Every 4 days, half of the volume was replaced with fresh medium. During the withdrawal process we avoided chopping the spheres.

### Growth rates

Isolated single neurospheres were placed in 96 multiwell plates to avoid neurospheres fusion. Multiwell plates were previously treated with Poly-Hema (Poly (2-hydroxyethyl methacrylate), P3932, Sigma) solution, to avoid neurosphere adhesion to the bottom of the wells. Neurospheres were maintained in the presence (CTR) or in the absence (MFM) of growth factors for 14 days, and the diameter of the neurospheres were measured every 7 days. For every group, 5 distinct neurospheres were measured. Growth rates values were subjected to ANOVA, followed by a Newman-Keuls post hoc test, significance set at p<0.05.

### Plating of neurospheres

Coverslips were coated with poly-l-lysine solution (Sigma, St. Louis, MO; 0.1 mg/mL in MilliQ water), washed twice with Milli Q water, and air dried in the hood; 30 µL of pre warmed laminin solution (kindly gifted by Prof. Helena Nader, Department of Biochemistry, UNIFESP) was added to each coverslip and incubated at 37°C for 30 min. Laminin was removed, and the coverslips were washed with 50 µL DMEM two times. 50 µL DMEM/2% B27 was added to each coverslip, and they were kept in the incubator until plating. Spheres were washed with 1 mL DMEM two times and plated in differentiation medium (70% DMEM/30% F12/2% B27). Approximately five spheres were plated onto each coverslip. The coverslips were kept in the incubator for 1 h to allow the cells to adhere. After adhesion, differentiation medium was added to fill the wells for *in vitro* differentiation studies. Whole spheres were allowed to differentiate for 7 days and then fixed in 4% paraformaldehyde (PFA).

### Preparation of neurospheres sections

Whole neurospheres from the control (CTR) and mitogen free medium (MFM) were cultured in suspension and taken from the flasks, washed with phosphate buffered saline (PBS) to remove the excess of culture medium and fixed in 4% PFA for 1 h at room temperature. Neurospheres were washed three times with PBS and then put in a PBS/10% sucrose solution for 1 h at 4°C, PBS/20% sucrose solution for 1 h at 4°C and, finally PBS/30% sucrose solution at 4°C overnight. Neurospheres were then mounted in Histo Prep (Fisher Scientific, Philadelphia, PA) and frozen in dry ice for 5 min, followed by freezing at −80°C overnight. Spheres were sectioned at 10 µm on a cryostat and placed on silanyzed slides (Superfrost slides, Fisher Scientific, Philadelphia, PA). Four slides were made for both hNPC groups and every 4th section was taken in each slide.

### Immunocytochemistry for β-tubulin III, GFAP, Gal-C and Nestin

Plated neurospheres and sections were blocked/permeabilized in 5% goat serum/0.1% Triton X-100 for 30 min (except for Gal-C, where detergent was not added). Primary antibodies for β-tubulin III (Sigma, St. Louis, MO; mouse IgG2b, 1∶200), glial fibrillary acidic protein (GFAP) (DAKO, Denmark; rabbit IgG, 1∶300), Gal-C (galactocerebroside C) (Chemicon, Temecula, CA; mouse IgG3, 1∶300), Nestin (Chemicon, mouse IgG, 1∶100) were added, and the cells were incubated overnight at 4°C. Cells were washed in PBS and incubated with the secondary antibodies Alexa 546 or Alexa 488 (Molecular Probes, Eugene, OR). After washing with PBS, DAPI solution (Sigma, St. Louis, MO; 0.3 µg/ml) was used as a nuclear stain. Cells were analyzed under a fluorescence microscope (Nikon, model Eclipse E600FM, Japan). Cell counts of β-tubulin III, GFAP, Nestin and DAPI were performed in sections that included the equatorial regions. After plating onto coverslips (n = 3; 5 neurospheres per coverslip) for 7 d, differentiated hNPC were analyzed qualitatively for the distribution, cell shape and organization. Approximately 7 fields of view, in the migration radius area of the neurosphere, were analyzed per coverslip. Higher magnification images of the differentiated cells were used to access neurite extension after plating and migration, considering the distances between the cell bodies and the end of the longer processes found (n = 15). The percentages were generated as the number of immunolabeled cells (GFAP, Nestin and β-tubulin III positive cells) divided by the total number of nuclei (DAPI stained) within cryosectioned spheres (n = 10). To verify the presence of GFAP and β-tubulin III co-labeling, double stained sections were analyzed in a confocal microscope (Zeiss, Axiovert 100M, LSM 510 software). Data were analyzed using the t-Student-test (significance set at p<0.05).

### Cell migration analysis

Seven days differentiating neurospheres were analyzed to measure cell migration outside neurosphere. Images from migrating cells stained with 4′-6-Diamidino-2-phenylindole (DAPI) (Molecular Probes, Eugene, OR) were taken in 4× magnification and the distances between the center of the neurosphere and the migratory boundary were measured. The ratios between the geometric radius of the neurosphere and the measured distances were calculated for the CTR and MFM groups. These ratios were used to normalize the results obtained from neurospheres with distinct sizes. Data were analyzed using the t-Student-test (significance set at p<0.05).

### BrdU incorporation

BrdU (0.2 µM) was added to the medium for 14 h to the CTR and MFM groups and, after this period, neurospheres were sectioned. Neurospheres sections were incubated in HCl 1.5M for 30 min. under gentle shaking, washed 3× 10 min. in PBS and blocked in 5% NGS/0.1% TritonX-100/PBS. Then they were incubated with anti-BrdU (Accurate Chemical & Scientific Corporation, Westbury, NY) for 2 h and washed three times in PBS. Cells were incubated with the secondary antibody (Alexa 488, Molecular Probes, Eugene, OR) for 1 h and washed three times in PBS. DAPI solution was used as a nuclear stain. Cells were analyzed under a fluorescence microscope (Nikon, model Eclipse E600FM, Japan). BrdU data were analyzed using the t-Student-test (significance set at p<0.05).

### TUNEL (Terminal deoxynucleotidyl transferase-mediated biotinylated UTP Nick End Labelling)

In situ cell death detection kit (Roche catalog # 1-684-795) labels apoptotic cells, based on labelling of DNA strand breaks, with fluorescein (green fluorescence). Cultured cells were fixed with 4% paraformaldehyde in PBS for 10 minutes, washed 3 times with PBS and blocked/permeabilized in 5%NGS+0.2%TX-100 in PBS for 15 minutes at room temperature. Cells were washed one time with PBS and the enzyme and nucleotide mix were added (Enzyme-TdT and Label-nucleotide) and incubated for 10 minutes at 37°C. Cells were washed 3 times with PBS and the nuclei were labelled with DAPI for 5 minutes. Cells were washed with PBS, mounted and analyzed under a fluorescence microscope (Nikon, model Eclipse E600FM, Japan). TUNEL data were analyzed using the t-Student-test (significance set at p<0.05).

### Cell Counting for BrdU and TUNEL

The percentages of BrdU and TUNEL positive cells were generated as the number of immunolabeled cells divided by the total number of cells (DAPI stained) within cryosectioned spheres. All data are expressed as mean±standard error.

### RNA extraction and cDNA synthesis

Total RNA of M031 CX cell line was isolated by using TRIzol (Invitrogen, Carlsbad, CA) reagent according to manufacturers' protocol. Total RNA concentration and integrity were determined by spectrophotometer readings at absorbance 260 nm and 280 nm and by observation of the Ribosomal RNA bands in a 1% agarose gel electrophoresis respectively. First strand cDNA synthesis was performed using SuperScript™ II Reverse Transcriptase (Invitrogen, Carlsbad, CA) as suggested by the manufacturer using 5 µg of total RNA. To avoid DNA contamination the RNA were previously treated for 30 minutes at 37°C with 1 U RQ1 RNase-Free DNase (Promega Corporation, Madison WI) in presence of 20 U RNAseOUT™ (Invitrogen, Carlsbad, CA) RNAse Inhibitor and the DNase was inactivated by a 95°C incubation for 15 minutes and immediately chilled on ice. Resultant cDNA was then used for polymerase chain reaction (PCR) as described below.

### Quantitative real-time PCR

Expression analysis of mRNA was performed in the ABI PRISM 7700 *sequence detection system* (Applied Biosystem, Foster City, CA) using SYBR®-Green amplification detection system. Each reaction was performed in a final volume of 20 µL using cDNA reversed transcribed from 25 ng of the RNA, 10 µL of the SYBR®-Green Universal PCR Master Mix and 1 µL of each forward and reverse primers (10 µM each) shown below. We conducted the real time PCR reactions separately using the following temperature protocol: 50°C-2 min, 95°C-10 min, and 50 cycles of 95°C-15s and 60°C-1 min, followed by a dissociation curve protocol to check the specificity of the amplicon produced in each reaction. To check reaction efficiency, we previously run standard curves for each primer set and cDNA sample. As the efficiency of all reactions was approximately 100% (>95%), we calculated the relative expression of each gene by the equation: 2^(CqTBP-CqGene)^, where Cq is the mean quantification cycle value of two independent RT-PCR real-time reactions for the analyzed gene and TATA binding protein (TBP) as endogenous control. Primers forward and reverse, respectively, are represented in the 5′→3′direction. Data were analyzed using the t-Student-test (significance set at p<0.05).

Primers for human RNA analysis:

EGF: AACGCCGAAGACTTACCCAG and CTTATTACTGATGGCATAGCCC
FGF2: CTAACCGTTACCTGGCTATGA and TTCGTTTCAGTGCCACATACCA
IGF1: ATGCTCTTCAGTTCGTGTGTGG and CTGTCTGAGGCGCCCTCCGA
NT3: TTACAGGTGAACAAGGTGATGTCC and CTGGCAAACTCCTTTGATCC
PDGFa: CATTCGGAGGAAGAGAAGCATCG and CTGGTCTTGCAGACAGCGGG
PDGFb: TGAACATGACCCGCTCCC and GCGTCTTGCACTCGGCG
β-tubulin III: AGACCTACTGCATCGACAACGAGG and GCTCATGGTGGCCGATACCAGG
GFAP: AAGAGTGGTATCGGTCCAAGTTTG and CAGTTGGCGGCGATAGTCAT
TBP: CCCCATCACTCCTGCCACGCC and CGAAGTGCAATGGTCTTTAGGTC

## Supporting Information

Figure S1 Gene expression profile after growth factor starvation in mNPC. After 10 days growth factors withdrawal, mNPC decreased the expression of FGF-2 and GFAP and increased the expression of IGF-1 and PDGFb (t-Student-test, p<0.05). mNPC also showed a clear tendency of increasing in β-tubulin III expression. The values are presented as the mean of three independent experiments with the standard deviation. Primers for mouse RNA analysis: TBP: CCCTATCACTCCTGCCACACC and CGAAGTGCAATGGTCTTTAGGTC. β-tubulin III: AGACCTACTGCATCGACAATGAAG and GCTCATGGTAGCAGACACAAGG; EGF: CCAAACGCCGAAGACTTATCC and CTTATTACCGATGGGATAGCCC; FGF2: CCAACCGGTACCTTGCTATGA and TTCGTTTCAGTGCCACATACCA; IGF1: GCCACACTGACATGCCCAAG and TGCACTTCCTCTACTTGTGTTCTTC; NT3: TTACAGGTGAACAAGGTGATGTCC and CCGGCAAACTCCTTTGATCC; PDGFa: CATTCGCAGGAAGAGAAGTATTG and CTGGTCTTGCAAACTGCGGG; PDGFb: CCTGCTGCACAGAGACTCCGT and CTCCCTCGAGATGAGCTTTCC GFAP: AGGAGTGGTATCGGTCTAAGTTTG and CAGTTGGCGGCGATAGTCGT Nestin: TGACCATTTAGATGCTCCCCAG and GTCCATTCTCCATTTTCCCATTC;
(0.86 MB TIF)

Table S1 Primers used in Supplemental Figure S1, forward and reverse, respectively represented in the 5′→3′direction.
(0.05 MB DOC)
