# Supplementary figures and images for: Short-Term Withdrawal of Mitogens Prior to Plating Increases Neuronal Differentiation of Human Neural Precursor Cells
Source: PLoS One. 2009 Feb 27;4(2):e4642. doi: 10.1371/journal.pone.0004642 (PMC2646132; doi:10.1371/journal.pone.0004642)

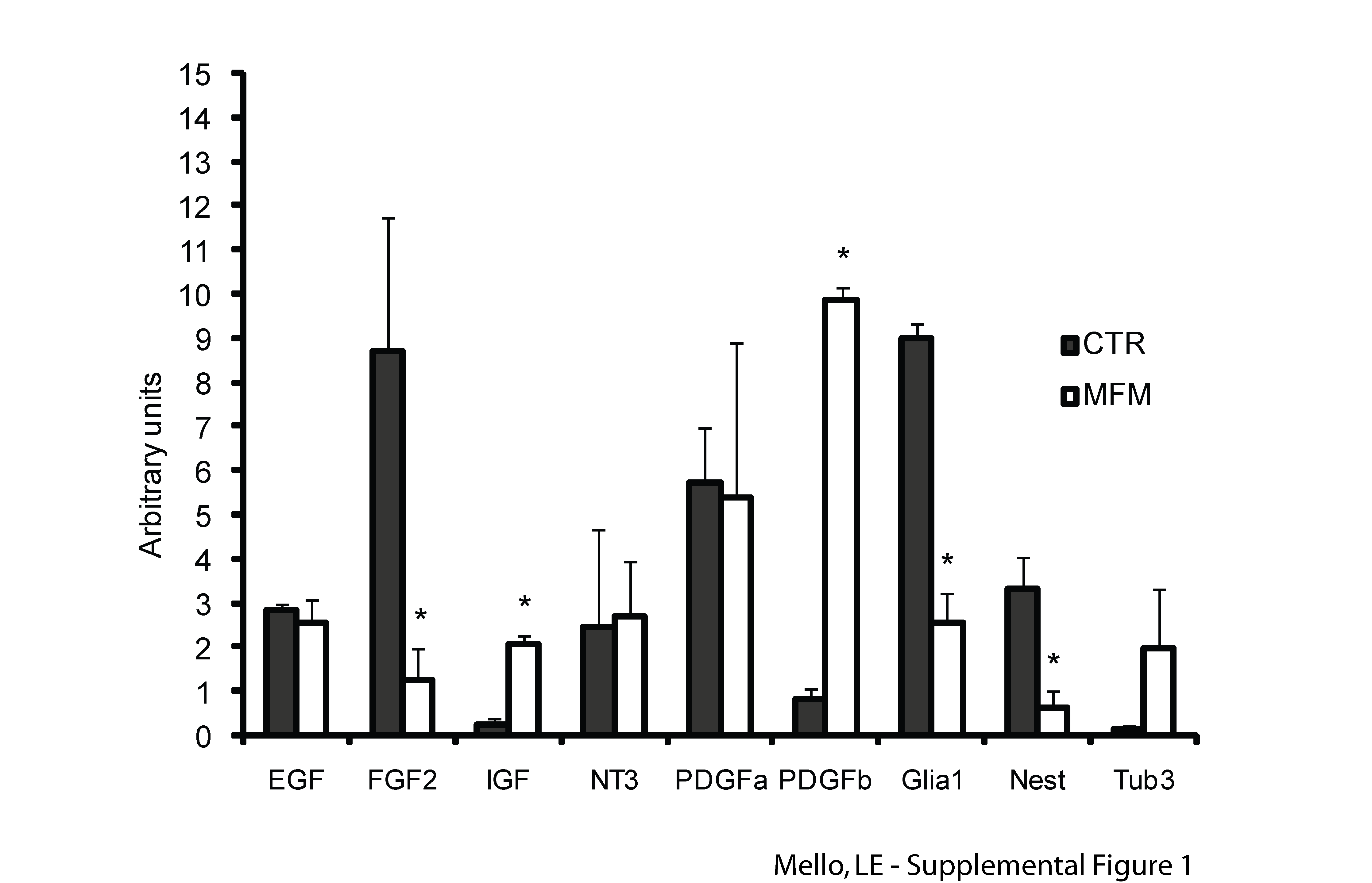

Supplement: Figure S1 — Gene expression profile after growth factor starvation in mNPC. After 10 days growth factors withdrawal, mNPC decreased the expression of FGF-2 and GFAP and increased the expression of IGF-1 and PDGFb (t-Student-test, p<0.05). mNPC also showed a clear tendency of increasing in β-tubulin III expression. The values are presented as the mean of three independent experiments with the standard deviation. Primers for mouse RNA analysis: TBP: CCCTATCACTCCTGCCACACC and CGAAGTGCAATGGTCTTTAGGTC. β-tubulin III: AGACCTACTGCATCGACAATGAAG and GCTCATGGTAGCAGACACAAGG; EGF: CCAAACGCCGAAGACTTATCC and CTTATTACCGATGGGATAGCCC; FGF2: CCAACCGGTACCTTGCTATGA and TTCGTTTCAGTGCCACATACCA; IGF1: GCCACACTGACATGCCCAAG and TGCACTTCCTCTACTTGTGTTCTTC; NT3: TTACAGGTGAACAAGGTGATGTCC and CCGGCAAACTCCTTTGATCC; PDGFa: CATTCGCAGGAAGAGAAGTATTG and CTGGTCTTGCAAACTGCGGG; PDGFb: CCTGCTGCACAGAGACTCCGT and CTCCCTCGAGATGAGCTTTCC GFAP: AGGAGTGGTATCGGTCTAAGTTTG and CAGTTGGCGGCGATAGTCGT Nestin: TGACCATTTAGATGCTCCCCAG and GTCCATTCTCCATTTTCCCATTC; (0.86 MB TIF) [file pone.0004642.s001.tif]
