# Supplementary material for: Short-Term Withdrawal of Mitogens Prior to Plating Increases Neuronal Differentiation of Human Neural Precursor Cells
Source: PLoS One. 2009 Feb 27;4(2):e4642. doi: 10.1371/journal.pone.0004642 (PMC2646132; doi:10.1371/journal.pone.0004642)
Supplement: Table S1 — Primers used in Supplemental Figure S1, forward and reverse, respectively represented in the 5′→3′direction. (0.05 MB DOC) [file pone.0004642.s002.doc]

**Supplemental Table S1**. Primers used in Supplemental Figure S1, forward and reverse, respectively represented in the 5´→3´direction:

β-tubulin III: AGACCTACTGCATCGACAATGAAG and GCTCATGGTAGCAGACACAAGG;

GFAP: AAGAGTGGTATCGGTCCAAGTTTG and CAGTTGGCGGCGATAGTCAT;

EGF: CCAAACGCCGAAGACTTATCC and CTTATTACCGATGGGATAGCCC;

FGF2: CCAACCGGTACCTTGCTATGA and TTCGTTTCAGTGCCACATACCA;

IGF1: GCCACACTGACATGCCCAAG and TGCACTTCCTCTACTTGTGTTCTTC;

NT3: TTACAGGTGAACAAGGTGATGTCC and CCGGCAAACTCCTTTGATCC;

PDGFa: CATTCGCAGGAAGAGAAGTATTG and CTGGTCTTGCAAACTGCGGG;

PDGFb: GAAAGCTCATCTCGAGGGAGG and GCGTCTTGCACTCGGCG;

Nestin: TGACCATTTAGATGCTCCCCAG and GTCCATTCTCCATTTTCCCATTC;

TBP: GAATCTTGGCTGTAAACTTGACCT and TCTTATTCTCATGATGACTGCAGCA.
